# Influence of Alcohol on Intracerebral Hemorrhage: From Oxidative Stress to Glial Cell Activation

**DOI:** 10.3390/life14030311

**Published:** 2024-02-27

**Authors:** Shaik Ismail Mohammed Thangameeran, Po-Kai Wang, Hock-Kean Liew, Cheng-Yoong Pang

**Affiliations:** 1 Institute of Medical Sciences, Tzu Chi University, Hualien 970, Taiwan; 106324122@gms.tcu.edu.tw; 2 Department of Anesthesiology, Hualien Tzu Chi Hospital, Buddhist Tzu Chi Medical Foundation, Hualien 970, Taiwan; 3 School of Medicine, Tzu Chi University, Hualien 970, Taiwan; 4 Department of Medical Research, Hualien Tzu Chi Hospital, Buddhist Tzu Chi Medical Foundation, No. 707, Section 3, Zhong-Yang Road, Hualien 970, Taiwan; 5 Neuro-Medical Scientific Center, Hualien Tzu Chi Hospital, Buddhist Tzu Chi Medical Foundation, Hualien 970, Taiwan; 6 PhD Program in Pharmacology and Toxicology, Tzu Chi University, Hualien 970, Taiwan

**Keywords:** alcohol consumption, intracerebral hemorrhage, alcoholism, alcohol intoxication

## Abstract

The intricate relationship between alcohol consumption and intracerebral hemorrhage (ICH) presents a nuanced field of study, especially concerning the dose-dependent impact on secondary brain injury (SBI). Recognizing the established risks associated with heavy drinking, this review delves deeper into the less understood territories of low to moderate alcohol consumption. By systematically analyzing recent studies, we uncover critical insights into how varying alcohol intake levels modulate ICH risk through mechanisms such as microglial activation, oxidative stress, and the protective potential of polyphenols. This analysis extends beyond the hypertensive effects of heavy alcohol use to explore the complex molecular pathophysiology involved in alcohol-related ICH. Our findings indicate that while heavy alcohol use unequivocally exacerbates ICH risk, moderate consumption and its associated polyphenols may offer neuroprotective effects against SBI, albeit within a finely balanced threshold. This review highlights the significant gaps in current understanding and underscores the urgent need for targeted research to elucidate these complex interactions. Through this comprehensive examination, we aim to inform more nuanced public health policies and intervention strategies, taking into account the diverse effects of alcohol consumption on ICH risk.

## 1. Introduction

Alcohol, a key part of human culture since ancient times, has various uses ranging from beverages to antiseptics and fuels. Different types of alcoholic beverages such as wine and beer, being the oldest and probably the most widely used drugs, were known for their therapeutic value in addition to the vital part they played in the daily life of people in ancient times. Ethanol, produced by fermentation, was consumed either in a diluted form or concentrated by distillation and these beverages were often considered divine, featuring in religious ceremonies, mythology, and social meals like the Greek symposia [1].

Despite its role in social events and its increased accessibility, alcohol abuse poses significant public health and economic challenges. In the exploration of alcohol’s impact on health, it is crucial to present a balanced view that encompasses both its potential benefits and risks. While moderate alcohol consumption has been associated with certain cardiovascular benefits, these effects must be critically weighed against the well-documented risks, especially concerning intracerebral hemorrhage (ICH). This includes the exacerbation of risk factors such as hypertension and the direct contribution to secondary brain injury (SBI) mechanisms. The nuanced nature of alcohol’s effects necessitates a careful examination of dose—response relationships and the differential impacts of consumption patterns [2,3,4]. Moderate alcohol consumption may offer neuroprotective benefits through mechanisms like oxidative stress reduction and improved vascular health, whereas heavy drinking exacerbates ICH risk factors [5].

Chronic heavy drinking is linked to increased hypertension and cardiovascular diseases [6,7,8], elevating blood pressure and disrupting blood coagulation [9]. Conversely, moderate drinking is often associated with positive cardiovascular effects [10,11,12] and could benefit cardiac health through certain physiological mechanisms [13,14]. Alcohol consumption also influences risk factors for ICH, such as hypertension, smoking, and the use of certain medications [15,16,17]. This review focuses on the intricate relationship between alcohol consumption and ICH.

## 2. Standard Drink of Alcohol

**Figure 1:**
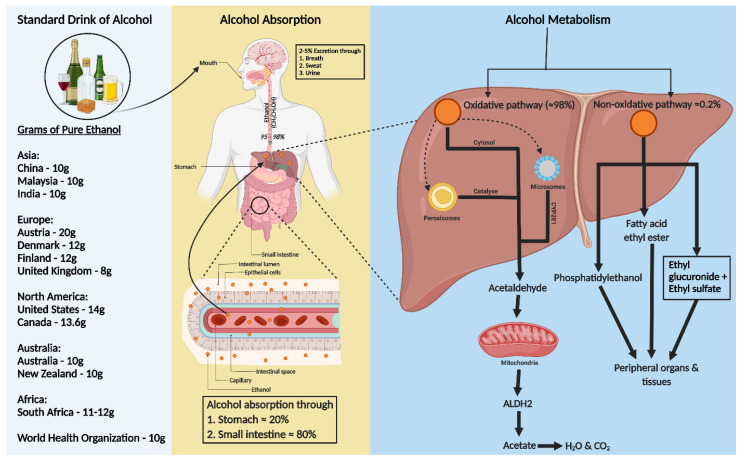
Consumption, absorption, and metabolism of alcohol. Consumption of alcohol (expressed as grams of ethanol per day) among different regions around the world is shown on the left of the figure. Alcohol is primarily absorbed in the small intestine (80%) and stomach (20%), with 2–5% excreted unchanged via breath, sweat, and urine. The rest, 95–98% of ethanol, is metabolized in the liver through two pathways: oxidative and non-oxidative. The oxidative pathway involves alcohol dehydrogenase (ADH) in the cytosol converting ethanol to acetaldehyde and further metabolism to acetate by aldehyde dehydrogenase 2 (ALDH2) in the mitochondria. Cytochrome P450 2E1 (CYP2E1) and catalase also play roles in ethanol metabolism, especially at higher consumption levels. In the non-oxidative pathway, ethanol forms fatty acid ethyl ester (FAEE) or phosphatidyl ethanol, catalyzed by FAEE synthase and phospholipase D. The metabolites from both pathways enter the circulation, impacting peripheral organs.

A standard alcohol unit, used to measure drinking consistently, varies globally. The World Health Organization (WHO) suggests that a standard drink contains 10 g of pure ethanol per day, though this varies (8–20 g) across countries (Figure 1). Kalinowski and Humphreys (2016) systemically reviewed government guidelines worldwide. The National Institute on Alcohol Abuse and Alcoholism (NIAAA) categorizes alcohol use as moderate (up to 1 drink daily for women and 2 for men), binge drinking (5 or more drinks for men and 4 for women, within about 2 h), and heavy alcohol use (more than 4 drinks per day or 14 per week for men and more than 3 drinks per day or 7 per week for women). In the US, a standard drink is defined as 14 g of pure alcohol [18].

## 3. Alcohol Absorption and Metabolism

Ethanol, a water, and lipid-soluble molecule, is easily absorbed through biological membranes. It is distributed quickly throughout the body after consumption, entering the bloodstream primarily via the stomach (20%) and small intestine (80%), with faster absorption in the intestine due to larger surface areas of villi and microvilli (Figure 1) [19,20]. Only 2–5% of alcohol is excreted unchanged through the lungs, skin, and kidneys [21]. Most ethanol is metabolized in the liver, through oxidative and non-oxidative pathways [22,23].

In the liver, alcohol dehydrogenase (ADH) converts ethanol to acetaldehyde, which is then rapidly changed to acetate by aldehyde dehydrogenase 2 (ALDH2) in the mitochondria [23,24]. This acetate enters the Krebs cycle, becoming water and carbon dioxide for elimination [25]. Cytochrome P450 monooxygenases (CYP2E1) and catalase in the peroxisome also contribute to oxidative metabolism, especially after heavy drinking [22,23,24,26,27].

Less than 0.2% of ethanol undergoes non-oxidative metabolism in the liver, forming ethyl glucuronide (EtG) and ethyl sulfate (EtS) [28]. Ethanol can also be metabolized to fatty acid ethyl ester (FAEE) via FAEE synthase and to phosphatidyl ethanol by phospholipase D [29] (Figure 1).

## 4. Alcohol Induced Hypertension

Research indicates that high alcohol consumption is linked to increased blood pressure, with clinical and preclinical studies confirming this correlation [30,31,32,33]. High alcohol intake activates the sympathetic nervous system and increases corticotropin-releasing factor secretion [34], impairs baroreceptor activity in the brainstem, and raises levels of cortisol and angiotensin, known blood pressure modulators [35,36]. Some studies illuminate the intricate interplay between alcohol consumption and vascular health. Chronic alcohol exposure significantly alters endothelial and smooth muscle function across various tissues, including the buccal mucosa and systemic circulation. This manifests as increased endothelin-1 levels, decreased nitric oxide synthase (NOS) activity, and direct effects on vascular smooth muscle, influencing vasodilation and blood pressure regulation. While moderate alcohol intake may confer some protective cardiovascular effects, chronic and heavy consumption exacerbate vascular dysfunction, highlighting alcohol’s dual role in cardiovascular health [37,38,39]. A review of 30 cross-sectional population studies found small but significant elevations in blood pressure in those consuming three drinks or more per day compared to nondrinkers [40].

Conversely, moderate alcohol consumption shows a potential effect of lowering blood pressure. Studies on individuals consuming up to two drinks per day did not find a correlation between reduced intake and decreased blood pressure, though cutting down alcohol did lower systolic and diastolic blood pressure [41]. The exact mechanism by which moderate alcohol consumption affects blood pressure remains unclear, suggesting a need for further molecular studies.

## 5. ICH

Spontaneous ICH, causing about 10 to 15% of all strokes with an incidence of 4.3 per 10,000 person-years, significantly impacts global health, particularly in low- and middle-income countries. In 2010, it affected 5.3 million people globally. The burden of both ischemic and hemorrhagic stroke has increased significantly in terms of the absolute number of incidents, deaths, and disability-adjusted life-years (DALYs) lost, with most of the burden in low-income and middle-income countries. High-income countries have seen a reduction in the incidence and mortality of these strokes, indicating the effectiveness of improved healthcare and preventive strategies. However, in low-income and middle-income countries, an increase in the incidence of hemorrhagic stroke and a non-significant increase in ischemic stroke have been noted [42].

ICH is a debilitating condition with only about 20% of survivors managing self-care six months post-ICH and 74% experiencing ongoing symptoms after a year. It has a high 30-day mortality rate of about 40%. ICH typically involves hematoma formation within the brain parenchyma, often in the putamen, and can affect areas like the cerebral lobes, basal ganglia, thalamus, brainstem, and cerebellum [43,44,45]. The condition encompasses two phases: an initial rupture of blood vessels, often due to hypertension or cerebral amyloid angiopathy, leading to primary brain injury from hematoma formation and expansion. Subsequently, SBI follows, involving hematoma clearance, immune response, neuroendocrine activation, and homeostasis response [46,47]. The severity of primary brain injury is linked to the site and volume of the hematoma, its expansion rate, and edema formation [43,48,49,50]. Large hematomas can be fatal due to increased intracranial pressure and potential brain herniation, restricting blood flow to certain brain regions [51].

## 6. SBI in ICH

**Figure 2:**
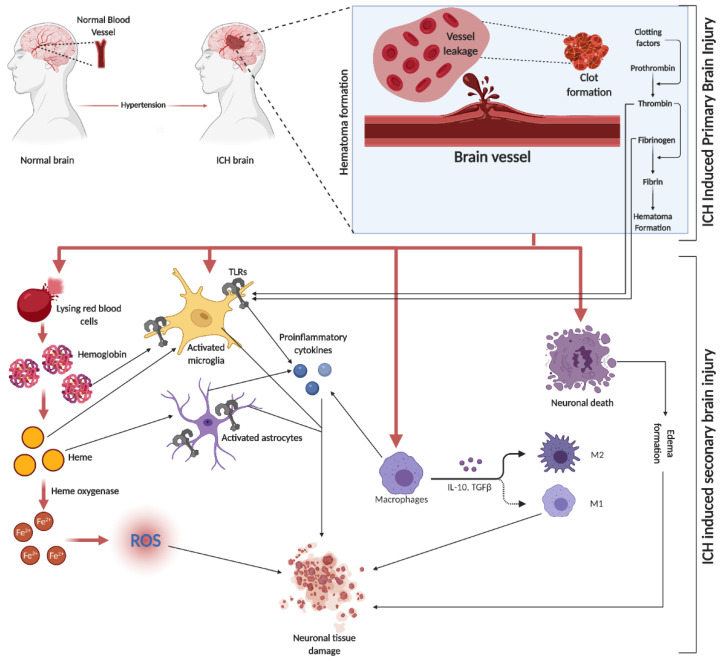
ICH is initiated by the rupture of blood vessels in the brain, leading to red blood cell (RBC) leakage and hematoma formation, facilitated by clotting factors like thrombin, fibrin, and fibrinogen. The subsequent SBI involves erythrocyte lysis, releasing Hb, which is broken down into heme and iron (Fe^2+^), triggering oxidative stress through various reactive oxygen species (ROS). This process activates microglia and astrocytes, with TLRs playing a role in their activation. The initial event causes direct neuronal damage and edema, further exacerbating tissue damage. Additionally, circulating macrophages infiltrate the injury site, differentiating into pro-inflammatory M1 and anti-inflammatory M2 macrophages, releasing a mix of cytokines.

SBI is a crucial factor in brain damage following ICH. It results from the volumetric compression of brain tissue and toxic blood components causing edema, blood–brain barrier (BBB) disruption, cell apoptosis, necrosis, and severe inflammation [52]. Hematomas disrupt neurons and glial cells, leading to neurotransmitter release and mitochondrial dysfunction. Coagulation products, particularly thrombin, initiate SBI, exacerbating blood-induced neurotoxicity [15,53,54]. This process activates microglia, subsequently releasing inflammatory cytokines, damaging the BBB, and causing neuronal apoptosis. Similarly, the breakdown of hemoglobin (Hb) activates astrocytes, contributing to neuronal damage through cytokine release [55]. Both microglia and astrocyte activation are mediated by toll-like receptors (TLR) [56] (a graphical representation of SBI events is depicted in Figure 2). In the evolving narrative of alcohol’s impact on ICH, emerging research delineates a J-shaped curve, signifying that while heavy consumption exacerbates ICH risk, moderate drinking may offer neuroprotection by mitigating oxidative stress and inflammation. This dual nature underscores the complexity of alcohol’s effects on SBI, suggesting a critical need for nuanced public health guidelines and personalized clinical strategies [57].

The relationship between alcohol consumption and ICH presents a complex interplay of factors that influence the onset and progression of this critical condition. Notably, the role of alcohol, ranging from moderate to heavy consumption, delineates varying paths of influence through oxidative stress, neuroendocrine disruptions, and neuroinflammatory responses leading to SBI. While the body of evidence, predominantly sourced from rodent models, offers significant insights into the molecular mechanisms underpinning these effects, the direct translatability of these findings to human pathophysiology remains a subject of considerable debate. This review acknowledges the indispensable value of animal studies in advancing our understanding while simultaneously highlighting the critical need for caution in extrapolating these results to human conditions. The inherent differences in metabolism, neuroanatomy, and the complexity of human behavior necessitate a nuanced approach to interpreting these findings, underscoring the imperative for further research that bridges this translational gap.

## 7. Role of Alcohol on SBI-Induced Toxic Products

Following ICH, there is an active clearance of red blood cells (RBCs) by microglia, macrophages, and infiltrating macrophages. RBCs break down into Hb, heme, and iron, with mechanisms like haptoglobin (Hp) binding to Hb to form a less toxic complex, aiding in preventing brain damage. Vascular injury during also leads to thrombin generation, contributing to clot formation and affecting microparticles from platelets and endothelial cells [15,54,56,58,59].

Studies have shown that alcohol consumption influences factors like fibrinogen and Hp, which are both primarily synthesized by the liver. Moderate alcohol intake (15.4 g/day) is associated with reduced fibrinogen levels, potentially lowering clot density and influencing cardiovascular health, particularly hypertension [60]. Similarly, moderate alcohol consumption correlates with lower Hp levels and can decrease fibrinogen levels in individuals post-exercise [61]. Additionally, chronic alcohol exposure in mice downregulated heme oxygenase (HO)-1, a key enzyme in heme catabolism during erythrocyte destruction [62].

## 8. Effect of Alcohol on Mitochondrial Dysfunction and Oxidative Stress in Brain Injury

Mitochondria, crucial in cellular function, regulate the balance between pro-oxidants and antioxidants, calcium homeostasis, ATP production, and stress responses [59]. After ICH, SBI involves toxic product release, leading to oxidative stress and cell injury. This process includes the breakdown of Hb into products like heme and iron. These products then produce hydroxyl radicals through the Fenton reaction, which exacerbates oxidative damage [63,64].

Oxidative stress, characterized by an overproduction of reactive oxygen species (ROS), surpasses antioxidant defenses, causing cellular damage. Antioxidants like superoxide dismutase (SOD) and glutathione peroxidase (GPx) decrease in models, indicating an imbalance in ROS and antioxidant production [65,66,67,68]. Microglial activation and iron toxicity from injury lead to lipid ROS accumulation and ferroptosis cell death, with proinflammatory cytokines further inducing ROS through enzymes like nicotinamide adenine dinucleotide phosphate (NADPH) oxidase [52,69,70,71,72]. SBI triggers mitochondrial dysfunction, with calcium and iron overload leading to increased ROS and apoptosis. Increased matrix metalloproteinases are associated with oxidative stress and BBB disruption [73,74,75,76,77]. Alcohol consumption, especially chronic heavy use, further exacerbates mitochondrial dysfunction and oxidative stress, impacting bioenergetics and increasing cell apoptosis [78,79,80].

Studies have shown varying effects of alcohol on ICH. Moderate alcohol preconditioning reduced hematoma volume, blood pressure, and oxidative and ER stress in a rat model, whereas high levels exacerbated these conditions [5]. Ethanol exposure was found to induce mitochondrial dysfunction and apoptotic gene regulation in human platelets [81]. These findings highlight the complex role of alcohol in mitochondrial function and oxidative stress in the context of ICH.

## 9. Effect of Alcohol on Neuroendocrine Axis Activation

Following ICH, the body activates the hypothalamic–pituitary–adrenal (HPA) axis and the sympathetic nervous system (SNS) to manage the stress induced by the injury. This response, involving the release of inflammatory cytokines, creates a feedback mechanism that further stimulates the HPA axis and SNS [82,83,84,85,86].

However, chronic alcohol consumption can impair HPA axis function. Studies have shown that mild alcohol intoxication inhibits the HPA axis response to cortisol [87,88], and active alcoholics exhibit compromised HPA axis activity under basal conditions [88]. Heavy and hazardous drinkers also have diminished cortisol reactivity compared to light or social drinkers [88,89,90]. Even in zebrafish larvae, 1% ethanol exposure led to increased HPA axis hormones [91]. This impairment suggests that heavy alcohol intoxication could hinder the necessary HPA axis response during CNS injuries. Conversely, chronic alcoholics exhibit increased SNS activity, with acute rises in blood alcohol levels also showing heightened SNS activity [92,93].

## 10. Alcohol and Activation of Complement System during SBI

Complement-mediated brain injury, particularly through the formation of the membrane attack complex (MAC), plays a key role in erythrocyte lysis, leading to delayed brain edema and inflammation after ICH [94]. MAC not only contributes to erythrocyte lysis but also influences cellular functions by releasing cytokines, ROS, and matrix proteins [95,96]. Components of the complement system, like C5a and C3a, are involved in leukocyte chemotaxis and microglial activation, respectively, enhancing inflammatory responses post-ICH [97,98]. The presence of complement C3d and C9 in peri-hematoma regions confirms the activation of the complement system in ICH [99].

Research on fetal alcohol spectrum disorders in rats shows that postnatal alcohol exposure results in the secretion of microglial exosome and the complement C1q, both of which are linked to ethanol-induced neuronal apoptosis and necrosis [100]. These findings underscore the need to understand the relationship between alcohol intoxication, the complement system, and ICH, especially regarding hematoma clearance, edema formation, and inflammation in the context of alcohol consumption.

## 11. Alcohol and Neuroinflammatory Mechanism Activated in SBI

Neuroinflammation in SBI after ICH is a complex and multifaceted process. It involves the activation of various immune cells, including microglia and astrocytes, and is marked by the release of cytokines and chemokines. These inflammatory mediators contribute significantly to brain tissue damage. The response is not just limited to local effects but also involves systemic reactions, including the neuroendocrine axis and the complement system, which further exacerbate the inflammation and subsequent neuronal damage [101,102,103].

**Figure 3:**
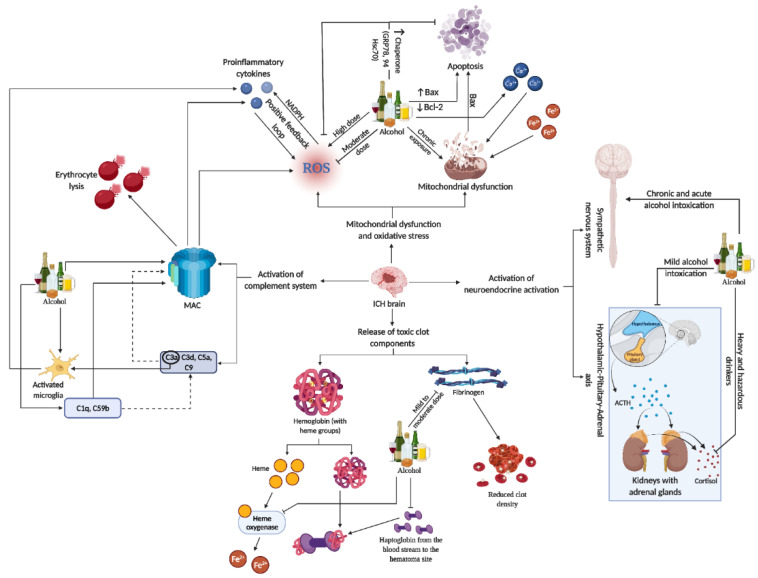
This representative figure delineates the multifaceted interactions between alcohol consumption patterns and brain injury subsequent to ICH. It maps out the cascade from alcohol-induced microglia activation and complement system engagement to the generation of ROS, mitochondrial damage, and apoptosis. It further illustrates systemic effects, including neuroendocrine responses, highlighting the extensive influence of alcohol on both brain pathology and peripheral organ systems in the context of ICH. For a detailed understanding, please refer to Section 11.

Recent studies have highlighted potential therapeutic targets within these neuroinflammatory pathways. Strategies to modulate this response, including the use of immunotherapies, are being explored. Therapeutic interventions aim to balance the inflammatory response, reducing the damaging effects while promoting beneficial repair mechanisms. This approach could significantly improve outcomes for patients suffering from ICH, as it addresses both the immediate and long-term consequences of the inflammatory response in the brain [101,104] (Refer to Figure 3 for the representative figure of the multifaceted effect of alcohol on ICH components).

### 11.1. Influence of Alcohol on Microglial Activation and Polarization

Microglia, the central nervous system’s resident macrophages, are activated by the release of toxic components from erythrocyte lysis following a hematoma, primarily through TLRs [105,106]. Their primary function is to clear hematoma debris, largely through pathways involving nuclear factor erythroid 2-related factor 2 (Nrf2) [107], peroxisome proliferator-activated receptor γ (PPARγ) [108], and interleukin-10 (IL-10) mediated regulation of CD-36 [109].

Microglia polarization occurs post-activation, differentiating into pro-inflammatory M1 microglia, which produce IL-6, IL-1β, TNF-α, and increase ROS, and anti-inflammatory M2 microglia, which release cytokines like IL-10 and IL-4, promoting nerve repair through neurotrophic and growth factors [110,111].

In a study using a four-day binge alcohol exposure model where rats received ethanol (5 g/kg), there was a notable increase in both M1 and M2 microglia populations in the hippocampus and entorhinal cortex. The rise in the M1 microglial phenotype was marked by increased expression of surface markers like MHC-II, CD86, and CD32, while the M2 phenotype was indicated by elevated CD206 levels [112]. Ethanol exposure in primary rat and mouse microglial cultures activated microglia and significantly increased proinflammatory cytokines like TNF-α and IL-1β [113]. In TLR4 knockout mice, ethanol exposure was linked to increased IBA1 (a microglial marker) immunoreactivity, with binge or chronic ethanol drinking also raising IBA1 levels [113]. Acute ethanol doses (50 mM) induced TLR4 and TLR2 interaction, releasing inflammatory mediators in microglial cells [113,114,115]. Furthermore, ethanol treatment was found to alter microglial morphology through NF-κB signaling, NADPH oxidase activity, and ROS production [115,116].

### 11.2. Influence of Alcohol on Astrocyte Activation

Astrocytes are crucial glial cells in the brain, involved in various functions including nutrient metabolism, neurogenesis, and maintaining the BBB. They contribute significantly to brain homeostasis and influence neuronal activity. Astrocytes are also involved in the pathophysiology of several neurodegenerative diseases, such as Alzheimer’s and Huntington’s, where their altered functions can exacerbate disease progression [117,118]. Astrocytes have a significant role in the body’s response to ICH. They express TLRs and release cytokines and chemokines upon activation, a process that becomes particularly prominent in response to brain injuries like ICH. These cells are crucial in mediating the inflammatory response during ICH, where the disruption of the BBB is a key event. This BBB disruption enables peripheral macrophages and neutrophils to infiltrate the injury site, exacerbating the condition. Astrocytes get activated during hematoma lysis by thrombin and hemoglobin through protease-activated receptors (PARs), contributing to an increase in lesion volume [118].

Intermittent heavy ethanol exposure in adolescent rats significantly affects astrocytes in the hippocampus. This exposure leads to increased activation of astroglial hemichannels and pannexons, contributing to neuroinflammation and altered astrocyte arborization. The study demonstrates that ethanol exposure increases the opening of connexin 43 (Cx43) hemichannels and pannexin-1 (Panx1) channels in astrocytes, which correlates with elevated levels of proinflammatory cytokines like IL-1β, TNF-α, and IL-6 in the hippocampus. The findings suggest that these changes in astrocyte activity and neuroinflammation could contribute to the neurotoxic effects of adolescent alcohol consumption [119]. High doses of ethanol elevate the number of GFAP+ astrocytes in the rat cerebral cortex [120] and increase astrocyte density in the prelimbic cortex of alcohol-preferring rats [121]. Astrocytes, when exposed to ethanol, exhibit significant changes in their inflammatory response mechanisms. This exposure leads to the induction of inducible nitric oxide synthase (iNOS) and the upregulation of cyclooxygenase-2 (COX-2). These molecular alterations are not merely indicative of an inflammatory state but are closely associated with the development of brain edema, a critical concern in neurological conditions. The activation of iNOS and COX-2 pathways suggests a direct link between ethanol exposure and the exacerbation of inflammatory responses in the brain, highlighting the potential risks associated with alcohol consumption, particularly in relation to neuroinflammation and brain edema [122,123]. Table 1 details the patterns and dosages of ethanol exposure, ICH models used, and their relation to SBI components.

### 11.3. Relationship between and Neurotrophic Factors (NTFs) and Influence of Alcohol on NTFs

Neurotrophic factors (NTFs) are crucial in regulating neuronal life and death, playing a key role in neurogenesis, synaptogenesis, and neuroprotection against apoptosis [124]. There are three major families of NTFs: the neurotrophin family including nerve growth factor (NGF), brain-derived neurotrophic factor (BDNF), neurotrophin-3 (NT-3), and NT-4; the glial-cell derived neurotrophic factor (GDNF) family; and the neuropoietic or interleukin-6 family. These NTFs are potential treatments for neurological diseases due to their regenerative capabilities [125,126,127,128].

After ICH, microglia and macrophages produce NTFs, particularly neurotrophins, to initiate repair. Studies have shown that BDNF can reduce hematoma volume, promote neural regeneration, and improve behavioral outcomes in models [129,130,131,132]. Similarly, NGF has been found to reduce inflammation and improve neurological function in patients [133,134]. The neurotrophic receptor p75 (p75NTR) is crucial in these processes, facilitating neurotrophins activities and mediating apoptosis. Models have shown that upregulated p75NTR is associated with increased apoptosis and inflammation, while its knockdown has protective effects [135,136].

Alcohol exposure, impacting both paternal and maternal sides, is known to significantly disrupt neurotrophin signaling pathways, crucial for neuronal development and regeneration. Prenatal alcohol exposure, in particular, has been shown to impair the synthesis of vital neurotrophins such as NGF and BDNF, critical for healthy brain development. This impairment is also associated with altered expression of neurotrophic receptors, leading to cellular damage. The subsequent increase in pro-inflammatory cytokines and ROS adds to the neurodevelopmental complications [137,138]. In adults with alcohol dependence, there is an observed disruption in the maturation process of BDNF, which is essential for neuronal survival, differentiation, and plasticity. The alteration in the conversion of pro-BDNF to mature BDNF and an increase in the levels of the p75NTR suggest a complex interplay between alcohol and brain chemistry that could have long-term implications on brain function and behavior [139]. Excessive alcohol consumption has also been linked to changes in BDNF signaling in brain regions associated with habit formation and addiction, such as the dorsolateral striatum. This change in signaling through p75NTR emphasizes the extensive and profound impact that alcohol has on neurotrophins and their receptors, which may contribute to the neuropathological changes observed in alcohol use disorders [140].

### 11.4. Beneficial Effects of Antioxidant Polyphenols

The growing body of research on plant-based compounds underscores their pivotal role in promoting human health and preventing chronic diseases. Recent studies have focused on the intricate ways in which dietary phytochemicals, such as polyphenols found in fruits, vegetables, and grains, interact with the gut microbiota, influencing metabolic, cardiovascular, and neurodegenerative disease outcomes [141,142]. These interactions have been shown to modulate inflammatory pathways, enhance antioxidant defense mechanisms, and improve gut barrier function, which are crucial for maintaining homeostasis and preventing disease progression [143].

Moreover, investigations into specific compounds like resveratrol and quercetin have illuminated their potential neuroprotective effects, particularly in the context of acute neurological injuries and chronic neurodegeneration. These natural antioxidants are found to mitigate oxidative stress, suppress inflammatory responses, and inhibit apoptosis in neuronal cells, offering promising therapeutic avenues for conditions such as ischemic stroke and intracerebral hemorrhage. The mechanisms behind these effects involve the activation of signaling pathways like Nrf2 and suppression of pro-inflammatory mediators, highlighting the complex interplay between dietary components and cellular health [144,145,146,147,148].

The capacity of polyphenols to modulate key signaling pathways has been linked to the reduction of oxidative stress, mitigation of inflammatory responses, and the promotion of neuronal survival and recovery post-stroke. These findings are supported by a range of studies utilizing animal models of middle cerebral artery occlusion (MCAO), which have been instrumental in delineating the mechanisms through which polyphenols exert their beneficial effects on the ischemic brain [149,150].

In addition to neuroprotection, the role of dietary antioxidants in combating oxidative stress and inflammation extends to the prevention and management of other chronic conditions, including cardiovascular diseases and cancer. The modulation of oxidative stress through dietary interventions is suggested to influence genetic and epigenetic mechanisms, with implications for disease risk and progression. This aspect of nutritional science opens new frontiers in personalized medicine, where dietary strategies could be tailored to individual genetic profiles to optimize health outcomes. The impact of these findings is profound, suggesting that incorporating a variety of phytochemical-rich foods into the diet could serve as a cost-effective and accessible strategy for enhancing public health [151,152,153].

Furthermore, the research underscores the therapeutic potential of polyphenols in the context of ischemic stroke, suggesting that dietary interventions enriched with polyphenolic compounds could offer a complementary approach to traditional stroke treatments. By focusing on the neuroprotective effects of polyphenols, studies have highlighted their ability to not only reduce the extent of brain damage following ischemia but also to enhance the recovery processes, thereby improving functional outcomes [150,154,155,156]. Despite the promising data, the translation of these findings into clinical practice necessitates further investigation into the optimal delivery methods, dosages, and combinations of polyphenols that maximize their efficacy and bioavailability. As the scientific community continues to explore the therapeutic avenues offered by polyphenols, their integration into preventive and treatment regimens for ischemic stroke represents a promising and innovative approach to reducing the burden of this debilitating condition.

### 11.5. Epidemiological Evidence Linking Alcohol and ICH

Epidemiological studies have established a clear correlation between factors like hypertension, alcohol use, smoking, and certain medications [41,42]. Alcohol, particularly, is a significant risk factor. Heavy alcohol consumption elevates blood pressure and impairs platelet aggregation, increasing the risk of hypertension [5,8]. Binge drinking, especially in young adults, is linked to increased stroke risk [157,158,159,160]. Studies like the ethnic/racial variations of intracerebral hemorrhage (ERICH) [161] and multicenter study on cerebral hemorrhage in Italy (MUCH)-Italy [162] specifically indicate that heavy drinking escalates risk, with the latter showing a significant impact on older individuals and an association with deep ICH. The ERICH study revealed that while heavy alcohol consumption increases risk, moderate consumption might be protective [161]. Similarly, one other study found that heavy drinking (>4 drinks/day) is more associated with hemorrhagic stroke types [163]. In contrast, moderate alcohol intake, in some studies, shows a reduced risk of cardiovascular disease and strokes [31,164,165]. However, this is contradicted by evidence linking moderate and heavy alcohol intake with increased spontaneous risk in hypertensive individuals. Alcohol’s role in pathophysiology involves exacerbating vascular damage and SBI [69,166,167,168,169,170]. Chronic alcohol consumption leads to hypertension and affects blood pressure [170,171]. Alcohol’s role in oxidative stress, endothelial dysfunction, and increased hematoma volume has been demonstrated in various animal models and clinical studies [172,173,174,175,176].

## 12. Conclusions

**Figure 4:**
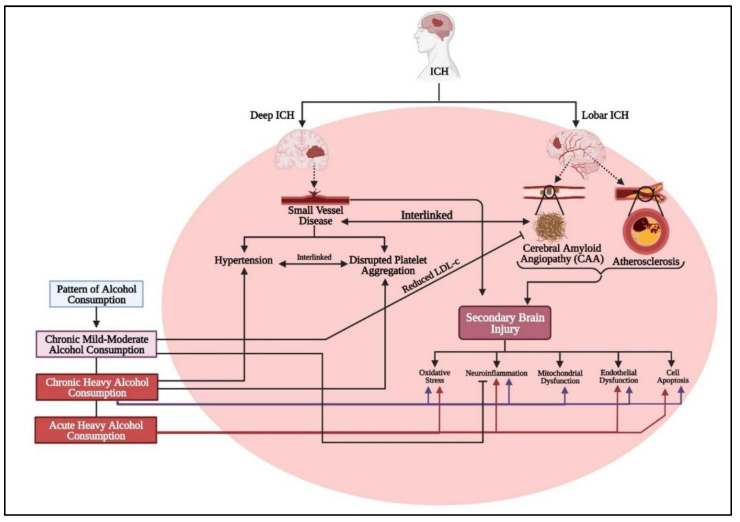
Alcohol consumption, categorized as chronic mild–moderate, chronic heavy, or acute heavy (binge drinking), has varying impacts on ICH. ICH is divided into deep ICH, primarily caused by small vessel disease due to hypertension and platelet dysfunction as well as lobar ICH, influenced by cerebral amyloid angiopathy (CAA) and atherosclerosis. Both types involve SBI mechanisms like oxidative stress, neuroinflammation, mitochondrial dysfunction, endothelial dysfunction, and cell apoptosis. Chronic heavy alcohol consumption exacerbates all these SBI mechanisms. Acute heavy consumption contributes to oxidative stress, neuroinflammation, endothelial dysfunction, and apoptosis but not mitochondrial dysfunction. In contrast, chronic mild–moderate consumption can be protective, reducing neuroinflammation and CAA by lowering low-density lipoprotein-cholesterol (LDL-c).

In this review, we have explored the intricate relationship between various alcohol consumption patterns and ICH. Chronic heavy, mild–moderate, and acute heavy drinking each uniquely impact ICH risk and severity. Alcohol’s role as a risk factor is multifaceted, intersecting with other ICH causes like hypertension, small vessel disease, cerebral amyloid angiopathy, and atherosclerosis (Figure 4). These conditions, influenced by lifestyle choices, are potentially worsened by alcohol, leading to ICH or aggravating its secondary brain injuries.

While rodent models have been instrumental in uncovering the potential pathways through which alcohol exerts its effects, the generalization of these results to human health requires careful consideration. The gaps in our understanding, particularly in the context of the dose-dependent effects of alcohol and the direct applicability of animal model findings to human ICH, highlight the urgent need for more comprehensive studies. Future research should aim not only to elucidate the nuanced effects of alcohol on ICH in humans but also to explore innovative models that can more accurately mirror human physiology and behavior. Addressing these research gaps will not only enhance our understanding of alcohol’s role in ICH but also pave the way for more effective prevention and treatment strategies that are grounded in a robust understanding of human biology.

The review underscores the need for deeper research into how different drinking behaviors affect ICH and its secondary outcomes. Understanding these dynamics is crucial for developing targeted prevention strategies and treatments. This area of study is vital for public health, especially considering the prevalent nature of alcohol consumption and the significant impact of ICH on individuals and healthcare systems. Further research could lead to more nuanced guidelines and interventions, ultimately reducing the incidence and severity of alcohol-related ICH.

## Figures and Tables

**Table 1:** Alcohol exposure and SBI Components. Upward arrow refers upregulation, downward arrow refers downregulation.

Relationship with SBI	SBI Components	Effects	Ref.
Direct relationship with neuroinflammation	↓ HO-1	Reduced heme catabolism thereby increasing the free heme in the brain parenchyma and exacerbating the injury	[59]
	↑ Intracellular Calcium	Mitochondrial dysfunction to neuronal apoptosis	[76,77]
	↓ ATP	Increased oxidative stress, mitochondrial dysfunction, and neuronal apoptosis	
	↑ MMP-9	BBB dysfunction	[78]
Direct relationship wtih neuroinflammation	↑ ROS-Increased oxidative stress	Increased neuronal tissue damage	[75]
	↑ Bax-Pro-apoptotic	Increased platelet apoptosis	[80]
	↓ Bcl-2-Anti-apototic		
Neuroendocrine Axis	↓ Cortisol Reactivity	Reduced HPA activity to mitigate the ICH injury	[88,89,90]
Complement System	↑ Complement C1q, C5b9 and MAC	Neuronal apoptosis and necrosis at the site of ICH injury	[100]
Inflammatory Mediators	↑ M1 and M2 microglial phenotypes	M1-Pro-inflammatory activities and M2-Anti-inflammatory activities	[112]
	↑ Pro-inflammatory cytokines (TNF-α, IL-1β)	Increased neuroinflammation	[113]
	↑ TLR-4 and TLR-2 interaction	Release inflammatory mediators	[115]
	↑ NF-κB signaling and NADPH oxidase	Increased ROS production and changes in microglial morphology thereby increasing neuroinflammation	[116]
	↑ iNOS ↑ COX-2	Activated immune response	[112]
	↑ Pro-inflammatory cytokines (TNF-α, IL-1β & IL-6), reduced chaperone proteins and diminished anti-inflammatory cytokines	Increased neuroinflammation	[79]
Direct relationship with neuroinflammation	↓ Fibrinogen	Reduces the clot density	[58]
	↓ Oxidative stress	Ameliorated neurological deficits	[79]
Neuroendocrine Axis	↓ HPA Acvitity	Compromised HPA activity dampens the stress mitigation caused by ICH	[86]
	↑ SNS Activity	Exerts already existing SNS activity thereby compromising the SNS activity needed to accord the stress caused by ICH	[92,93]
Inflammatory mediators and stressors	↓ ER stress	Increased ER homeostasis	[79]
	↑ Chaperone proteins (GRP78, GRP94 & Hsc70)	Reduced ER stress thereby reducing oxidative stress and neuronal apoptosis	
	↓ Pro-inflammatory cytokines (TNF-α, IL-1β & IL-6)	Reduced neuroinflammation	
	Restored anti-inflammatory cytokine (IL-10)		

## Data Availability

No new data were created or analyzed in this study. Data sharing is not applicable to this article.
